# Failure Patterns of Recurrence in Patients With Localized Esthesioneuroblastoma Following Surgery and Adjuvant Radiotherapy Without Elective Nodal Irradiation

**DOI:** 10.7759/cureus.46523

**Published:** 2023-10-05

**Authors:** Atsuto Katano, Masanari Minamitani, Shingo Ohira, Hideomi Yamashita

**Affiliations:** 1 Department of Radiology, The University of Tokyo Hospital, Tokyo, JPN; 2 Department of Comprehensive Radiation Oncology, Graduate School of Medicine, The University of Tokyo, Tokyo, JPN

**Keywords:** local control rates, disease-free survival, overall survival, retrospective analysis, recurrence patterns, elective nodal irradiation, adjuvant radiotherapy, recurrence, esthesioneuroblastoma

## Abstract

Background: Esthesioneuroblastoma (ENB), a rare malignancy arising from the olfactory epithelium, poses clinical challenges owing to its propensity for local invasion and recurrence. Its management typically involves surgical resection and adjuvant radiotherapy. However, debate persists regarding the optimal treatment strategy, particularly the use of elective nodal irradiation (ENI). This study aimed to investigate recurrence patterns in patients with localized ENB treated with surgery and adjuvant radiotherapy without ENI.

Methods: Our retrospective analysis included patients who underwent surgery followed by adjuvant radiotherapy for treatment of ENB between January 2011 and November 2022. Patients with incomplete data or who had received neoadjuvant radiotherapy were excluded. Patient characteristics, radiotherapy data (type, dose, and duration), and follow-up data were collected. Recurrence patterns were evaluated, and overall survival (OS), disease-free survival (DFS), and local control rates were determined using the Kaplan-Meier method.

Results: Twelve patients with ENB (median age, 56 years) were included. Most had stage C disease. The median radiation dose was 60 Gy, and the median treatment duration was six weeks. Only one death was confirmed during the observation period, and the five-year DFS rates were 64.3%. Local control was achieved in 11 patients, with only one experiencing local recurrence. Regional lymph node recurrence occurred in three patients and was successfully managed via neck dissection. The timing of recurrence varied, emphasizing the importance of long-term surveillance.

Conclusion: Adjuvant radiotherapy without ENI is a viable treatment option for ENB, resulting in favorable local control and OS outcomes. Regional lymph node metastases were observed but effectively managed via salvage therapy. Prospective studies with larger cohorts are warranted to confirm the effectiveness of this treatment strategy and to define optimal radiotherapy fields.

## Introduction

Esthesioneuroblastoma (ENB) originates from the olfactory epithelium and accounts for approximately 5% of all nasal cavity malignancies [1]. ENB exhibits a bimodal age distribution, with one peak in young adults and the other in individuals in their sixth and seventh decades of life [2]. Although rare, ENB presents significant clinical challenges owing to its proclivity for local invasion, recurrence, and distant metastasis.

The management of ENB has evolved over time, and a multimodal approach comprising surgical resection and adjuvant radiotherapy has been primarily adopted [3]. An analysis of 511 ENB cases from the Surveillance, Epidemiology, and End Results database demonstrated that better overall survival rates were achieved with combined surgery and radiotherapy (73%) than with surgery (68%) or radiotherapy (35%) alone [4]. Adjuvant radiotherapy is commonly integrated into ENB treatment protocols to enhance local control and reduce the risk of recurrence. Nevertheless, debate persists regarding the optimal treatment strategy, especially the use of elective nodal irradiation (ENI) in patients with localized disease. ENI proactively irradiates uninvolved regional lymph nodes; however, its benefits in terms of local control and overall survival (OS) remain controversial.

This study aimed to identify the sites and patterns of recurrence in patients with localized ENB who underwent surgical intervention and adjuvant radiotherapy without ENI. The insights gleaned from our thorough examination of this patient cohort have the potential to guide treatment decisions and enhance outcomes for individuals grappling with this rare and complex malignancy.

## Materials and methods

In this study conducted at The University of Tokyo Hospital, Tokyo, Japan, we retrospectively analyzed patients pathologically diagnosed with ENB who underwent surgery followed by adjuvant radiotherapy between January 2011 and November 2022. In this study, we enrolled patients who met the specified inclusion criteria, which included: (i) a confirmed diagnosis of ENB through histological examination, (ii) being treated with radiotherapy following surgery, (iii) the absence of distant metastasis, and (iv) no prior history of radiotherapy in the para sinus region. We retrospectively gathered patient data from our institution's medical records, and the Eastern Cooperative Oncology Group Performance Status scale was used to assess patients' performance status. Patients with incomplete data or who had received neoadjuvant radiotherapy were excluded. This study was approved by the Research Ethics Committee of The University of Tokyo (approval number: 3372-6).

**Figure 1:**
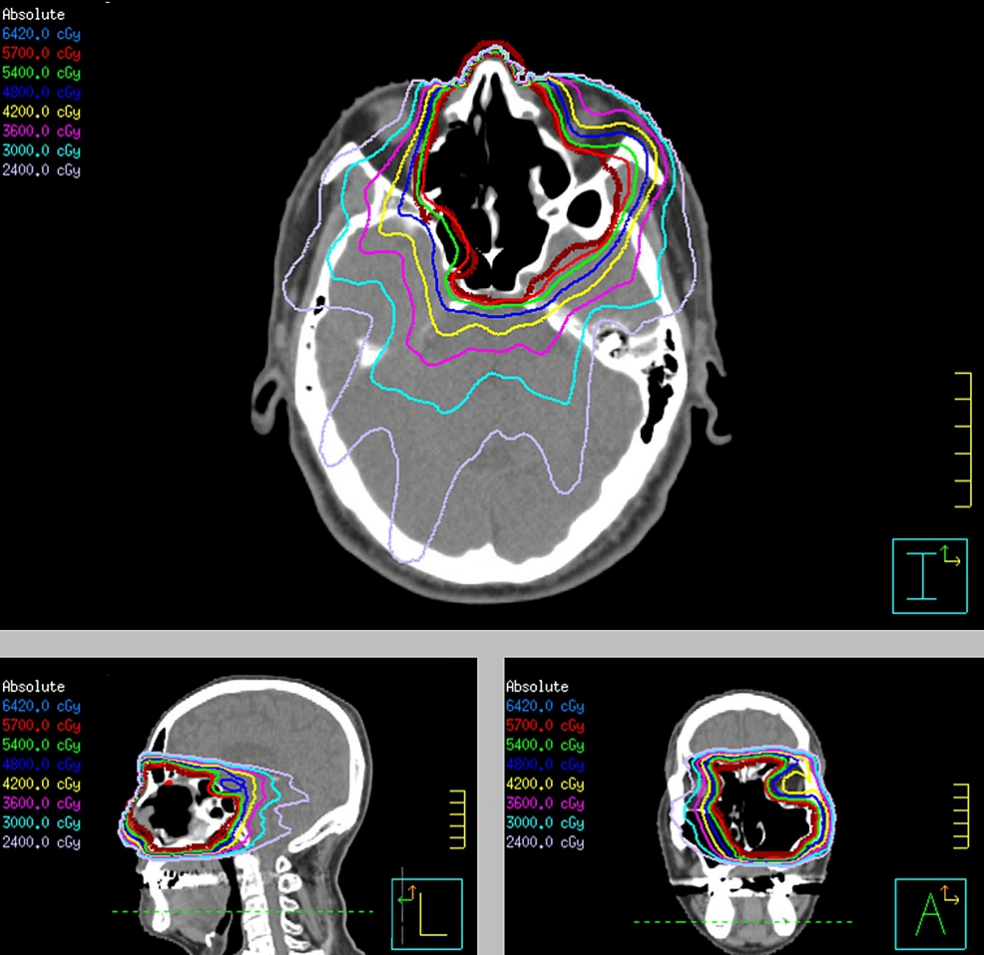
Treatment plan of adjuvant radiotherapy for esthesioneuroblastoma Adjuvant radiotherapy dose distribution for esthesioneuroblastoma. The planning target volume is delineated by a thick brown line. Transverse, sagittal, and coronal sections are shown.

The radiotherapy techniques used were intensity-modulated radiotherapy (IMRT) and three-dimensional conformal radiotherapy (3D-CRT). All patients received five daily fractions per week. Adjuvant radiotherapy was administered using an Elekta Synergy linear accelerator (Elekta, Stockholm, Sweden) with a high agility head, high-resolution beam-shaping multi-leaf collimator system, and helical tomotherapy (Accuray Inc., Sunnyvale, California). All patients were set up in a supine position using a custom-made thermoplastic mask. Following this, a computed tomography (CT) simulation was carried out with a slice thickness of 2 mm scanning the head. The adjuvant radiotherapy targeted the tumor bed or ipsilateral para sinus cavity, and the prescribed dose line was aimed to encompass more than 95% of the planning target volume, while adhering to the dose tolerance limits of critical neurological organs at risk (Figure 1).

Data were analyzed using R statistical programming language (R Foundation for Statistical Computing, Vienna, Austria). Survival was assessed using the Kaplan-Meier method. The primary aim of this study was to examine the recurrence patterns of the patients with ENB who underwent surgery followed by adjuvant radiotherapy. Also analyzed were OS, disease-free survival (DFS), and the rate of local control. OS was defined as the duration from diagnosis to death from any cause, and DFS was defined as the duration from diagnosis to the first recurrence or death from any cause.

## Results

Our study comprised 12 patients with ENB. The median age was 56 years (range, 29-71 years), and 11 patients (91.7%) were male (Table 1).

**Table 1:** The characteristics of the patients ECOG: Eastern Cooperative Oncology Group; 3D-CRT: three-dimensional conformal radiation therapy; IMRT: intensity-modulated radiation therapy

Variable		Value
Age (years), median (range)		56 (29–71)
Sex, n (%)		
	Male	11 (92%)
	Female	1 (8%)
ECOG performance status, n (%)		
	0	10 (83%)
	1	2 (17%)
Kadish group, n (%)		
	A	3 (25%)
	B	2 (17%)
	C	7 (58%)
Initial/Recurrent, n (%)		
	Initial	10 (83%)
	Recurrent	2 (17%)
Radiotherapy modality, n (%)		
	3D-CRT	2 (17%)
	IMRT	10 (83%)
Dose/ fractions, n (%)		
	50 Gy/25 fractions	1 (8%)
	60 Gy/30 fractions	10 (83%)
	68 Gy/34 fractions,	1 (8%)

According to the modified Kadish staging system [5], three patients had stage A disease, two had stage B disease, and seven had stage C disease. Two of the seven patients with stage C disease had undergone curative intent surgery before the current surgery and were considered recurrent cases. Ten patients were treated with IMRT and two with 3D-CRT. The median radiation dose was 60 Gy (range, 50-68 Gy), and the median treatment duration was six weeks (range, 5-8 weeks). Ten patients received 60 Gy in 30 fractions. One of the remaining patients received 50 Gy in 25 fractions to strictly adhere to the optic nerve dose constraints when using 3D-CRT. The final patient had a severe infection following administration of 40 Gy; after a respite from radiation treatment, this patient received an additional 28 Gy, resulting in a total dose of 68 Gy in 34 fractions.

**Figure 2:**
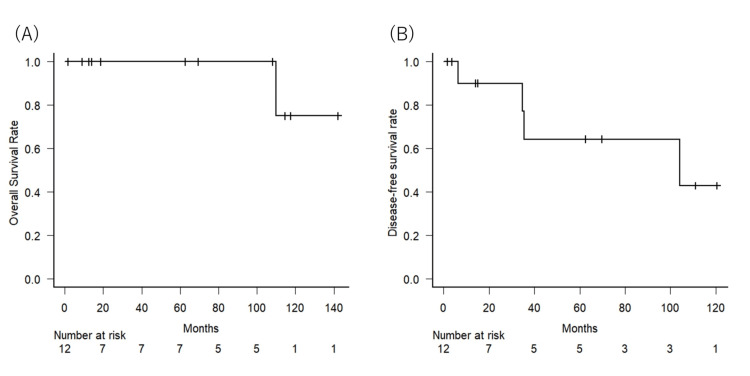
Kaplan–Meier curves for overall survival (A) and disease-free survival (B), n=12

The median follow-up time was 66 months (range, 1.6-142 months). No patients died during the first five years of follow-up for the entire cohort. During the follow-up period, four patients experienced disease recurrence. The DFS rates at five years were 64.3% (95% CI, 24.5-87.1 % ). Local control was achieved in 11 patients, and the six-year local control rate was 85.7% (95%CI, 33.4-97.9). The Kaplan-Meier curves for OS and DFS are presented in Figure 2.

Four patients experienced any kind of disease recurrence (Table 2).

**Table 2:** Description of the clinical profiles and the treatment approach for the four patients who encountered recurrence. PORT: postoperative radiotherapy; RT: radiotherapy; ND: neck dissection; RP: retropharyngeal lymph node; SRS: stereotactic radiosurgery

Age (years)	Sex	Kadish group	Recurrence	Months from PORT	Location	Salvage therapy
59	Male	C	1st	35	Right cervical node (Level II)	Right ND
			2nd	76	Dural metastasis	SRS
			3rd	103	Dural metastasis	SRS
			4th	114	Left cervical node (Level II)	Left ND
65	Male	C	1st	104	Right cervical node (Level Ib, II), left RP	Bilateral ND, definitive RT for RP
58	Male	C	1st	35	Local	Surgery
			2nd	55	Right cervical node(II, III)	Right ND
			3rd	81	Masseter metastasis	Surgery
			4th	82	Bones	BSC
49	Male	C	1st	6	Left cervical node (Level Ib)	Left ND

In three of the four patients, the first recurrence involved the regional lymph nodes. All patients with lymph node recurrence underwent neck dissection. One of the three patients also received definitive radiotherapy (60 Gy in 30 fractions) for retropharyngeal lymph node recurrence; these lymph nodes were not irradiated postoperatively in this patient. Some patients experienced repeated recurrence, and the sequence of recurrence and salvage therapy is summarized in Table 2. No grade 3 or higher adverse events were reported during both the acute and late phases of the study. In the acute phase, all patients experienced grade 1-2 mucositis and dermatitis. The most common late-phase adverse event was grade 1-2 sinus disorders, observed in eight patients.

## Discussion

Four (33.3%) patients in our study cohort experienced disease recurrence during the follow-up period. The distribution of the initial recurrence sites was notable: the regional lymph nodes in three patients and the primary tumor in one patient. Although regional lymph node recurrence was observed to some extent in our study, it was successfully salvaged via neck dissection in all patients. The OS rate obtained in this study (75% at 10 years) was comparable to those reported in previous studies. In an analysis using the National Cancer Database to assess the outcomes of 1,225 patients with ENB, the five-year OS rate was 76.2% [6]. Among 138 non-metastatic ENB cases from 1984 to 2018, the five-year OS rate was 69.6% [7]. Another important consideration is the timing of recurrence. In our study, the timing ranged from six to 104 months (median, 35 months). This variability highlights the need for long-term surveillance and follow-up, even years after the completion of adjuvant radiotherapy.

The dose and duration of radiotherapy used in our study were consistent with those used in previous studies of ENB. Ozsahin et al. suggest that postoperative radiotherapy should be performed using at least 54 Gy in patients with R0 or R1 surgical resection margins [8]. In a retrospective review by investigators at the MD Anderson Cancer Center, ENI reduced nodal recurrence following postoperative radiotherapy [9]. The results of that study indicate that adjuvant radiotherapy can significantly improve locoregional control in patients with ENB; however, no significant improvements in the OS rate were observed. Demiroz et al. examined the risk of cervical lymph node metastases in 26 patients with ENB who received treatment without ENI at the University of Michigan between 1995 and 2007 [10]. Regional failures were observed in seven (27%) patients, and the local relapse-free survival rate was notably low. In our study, the failure rate was similar; however, the recurrences were well managed with neck dissection, and the OS rate was favorable.

The efficacy of systemic therapy for managing recurrent or refractory metastatic ENB is a topic of ongoing investigation. Turano et al. reported that platinum-based chemotherapy (cisplatin, etoposide, doxorubicin, ifosfamide, and vincristine) provided pain relief and maintained stability for 24 months [11]. Wick et al. demonstrated successful management and enhanced quality of life in a patient with metastatic ENB treated with temozolomide [12]. However, no specific chemotherapy regimen has been definitively documented. A systematic review of 118 patients from 48 studies highlighted the use of platinum-based regimens but did not establish their survival advantage over other regimens [13]. A phase 2 trial of Bintrafusp alfa, a bifunctional protein targeting transforming growth factor-beta and programmed cell death ligand 1, for the treatment of metastatic ENB, is ongoing [14]. 

Our study has some limitations. Owing to its retrospective nature, it was susceptible to selection bias and confounding variables. Additionally, the small sample size restricts the generalizability of our findings. Prospective studies with larger cohorts and longer follow-up durations are necessary to validate the efficacy of adjuvant radiotherapy without ENI in ENB patients. Song et al. also reported that regional failures could be resolved using salvage treatment; then ENI is not necessary for clinically non-lymph node metastatic cases [15].

## Conclusions

Our retrospective analysis revealed the treatment outcomes associated with adjuvant radiotherapy without ENI in the treatment of ENB. Our findings demonstrated promising results concerning local control and OS rates. Despite the identification of regional lymph node recurrences in certain cases, these cases were well addressed through salvage therapy. The treatment strategy of limiting the radiation field to the vicinity of the tumor bed or ipsilateral nasal sinus cavity without ENI was considered to be a potentially useful radiotherapy strategy option. Further research is warranted to define optimal radiotherapy fields and to confirm the effectiveness of this treatment strategy.
